# Distribution of diandric and digynic triploidy depending on gestational age

**DOI:** 10.1007/s10815-021-02202-4

**Published:** 2021-05-13

**Authors:** Diana Massalska, Katarzyna Ozdarska, Tomasz Roszkowski, Julia Bijok, Anna Kucińska-Chahwan, Grzegorz Mieczysław Panek, Janusz Grzegorz Zimowski

**Affiliations:** 1 Department of Gynecologic Oncology and Obstetrics, Centre of Postgraduate Medical Education, Czerniakowska 231, 00-416 Warsaw, Poland; 2 Department of Genetics, Institute of Psychiatry and Neurology, Sobieskiego 9, 02-957 Warsaw, Poland

**Keywords:** Diandric (paternal) triploidy, Digynic (maternal) triploidy, QF-PCR, MS-MLPA

## Abstract

**Purpose:**

To establish the distribution of diandric and digynic triploidy depending on gestational age.

**Methods:**

107 triploid samples tested prospectively in a single genetic department during a four-year period were analyzed for parental origin of triploidy by Quantitative Fluorescent Polymerase Chain Reaction (QF-PCR) (*n*=95) with the use of matching parental samples or by MS-MLPA (*n*=12), when parental samples were unavailable. Tested pregnancies were divided into three subgroups with regard to the gestational age at spontaneous pregnancy loss: <11 gestational weeks, 11–14 gestational weeks, and >14 gestational weeks.

**Results:**

Diandric triploidy constituted overall 44.9% (46.5% in samples miscarried <11 gestational weeks, 64.3% in samples miscarried between 11 and 14 gestational weeks, and 27.8% in pregnancies which survived >14 gestational weeks).

**Conclusions:**

The distribution of diandric and digynic triploidy depends on gestational age. The majority of diandric triploid pregnancies is lost in the first trimester of pregnancy. In the second trimester, diandric cases are at least twice less frequent than digynic ones.

## Introduction

Triploidy is a frequent genetic aberration affecting around 1–2% of all conceptions [1]. The prevalence of triploidy decreases dramatically with gestational age as the majority of cases is miscarried in early pregnancy [2, 3]. Triploidy results from an extra haploid set of chromosomes of paternal (diandric) or maternal (digynic) origin. Three different triploid karyotypes are possible: 69,XXX; 69,XXY; and 69,XYY. In case of more common karyotypes 69,XXX and 69,XXY, the extra chromosomal set may be of paternal or maternal origin and further investigations are necessary to detect parental contribution. 69,XYY is always of paternal origin and rarely occurs or is lost at preclinical stages of pregnancy [4].

The sonographic phenotype depends on the parental origin, which is an evidence for genomic imprinting [5]. Even though conventional karyotyping does not identify the parental origin of triploidy, the majority of cases diagnosed beyond 11 gestational weeks may be accurately classified as diandric or digynic based on ultrasound assessment [6, 7]. However, if miscarried at earlier developmental stages, further molecular testing is needed to establish the parental origin of triploid pregnancy, which is rarely conducted in routine practice [8]. The prevalence of diandric triploidy ranges between 20 and 85% in different studies [1, 4, 7, 9–15]. However, the distribution of diandric and digynic triploidy with regard to gestational age has not been accurately established to date.

The aim of this study was to establish the distribution of diandric and digynic triploidy depending on gestational age.

## Material and methods

### Tested samples

All 107 triploid samples collected prospectively and tested in the Genetic Department of the Institute of Psychiatry and Neurology in Warsaw in the years 2017–2020 were included in the study.

Gestational age of tested pregnancies was established according to the last menstrual period in pregnancies <11 gestational weeks and corrected according to the first-trimester ultrasound results in pregnancies beyond 11 gestational weeks. All tested samples were divided into three subgroups with regard to gestational age at spontaneous pregnancy loss: <11 gestational weeks, 11–14 gestational weeks, and >14 gestational weeks. Viable pregnancies that were terminated beyond 14 gestational weeks were included into the third group. There was no pregnancy termination before 14 gestational weeks in our study group.

### Sample preparation and DNA extraction

In samples from invasive testing of viable pregnancies, DNA was extracted from 10 mg of the chorionic villi or amniocytes. The samples from pregnancy loss, obtained after prostaglandin administration and/or surgical evacuation, were examined under a microscope. Chorionic villi were dissected from blood clots and maternal decidua. DNA was extracted from 10 mg of the villi by DNA Xpure Cell&Tissue micro (A&A Biotechnology, Gdynia, Poland). Parental DNA for QF-PCR was extracted from peripheral blood lymphocytes (*n*=45×2) or buccal smears (*n*=50×2).

### Molecular testing

If parental DNA was available, fetal triploid samples with matching samples from both parents were tested for parental origin using Quantitative Fluorescent Polymerase Chain Reaction (QF-PCR). 26 selected microsatellite sequences from chromosomes 13, 18, 21, X, and Y were amplified. Reaction products were separated by capillary electrophoresis (3130 Genetic Analyzer, Applied Biosystems, Waltham, MA, USA), and the results were analyzed by the use of GeneMapper Software 5.0 (Thermo Fisher Scientific, Waltham, MA, USA).

In case of unavailable parental DNA (*n*=12), MS-MLPA was performed to establish the parental origin of triploidy according to the protocol described previously [16].

### Data analysis

Descriptive statistical analysis was performed using Microsoft Office Excel 2007.

### Ethical approval

The study was approved by the Bioethics Committee of the Centre of Postgraduate Medical Education in Warsaw (Approval No. 82/PB/2017). Informed consent for molecular testing was obtained from all participants.

## Results

Tested samples were obtained by either invasive prenatal procedures performed in viable first- and second-trimester triploid pregnancies (chorionic villus sampling (CVS), *n*=13; amniocentesis (AC), *n*=17) in a single tertiary referral centre for fetal medicine in Warsaw (Department of Gynecologic Oncology and Obstetrics of the Centre of Postgraduate Medical Education) or by dilation and curettage in spontaneous pregnancy losses performed in various gynecologic departments (*n*=77) tested at parental request and cost. All invasive procedures were performed due to fetal anomalies consistent with triploidy after informed consent.

During the study period, triploidy was detected in 30 out of 2205 invasive procedures performed in the Department of Gynecologic Oncology and Obstetrics (1753 amniocentesis, 402 chorionic villus sampling, and 50 kordocentesis; 1.4% of tested pregnancies; 30/2205) and in 83 out of 736 samples from miscarried pregnancies tested for chromosomal aberrations in the Genetic Department of the Institute of Psychiatry and Neurology (11.3% of all tested samples; 83/736); out of which 77 samples were available for testing for parental origin of triploidy (92.8%; 77/83) and were included in this study. To our knowledge, no other case of triploidy during this study period was confirmed.

There were 107 fetal triploid DNA samples (69,XXX: *n*=52; 69,XXY: *n*=54; and 69,XYY: *n*=1) included in the study. The mean gestational age at testing was 12 weeks (*n*=107; range: 6–26 weeks; 16.1 gestational weeks (range: 12–26 weeks) in case of samples from invasive prenatal procedures; 10.1 weeks (*n*=77; range: 6–18 weeks) in case of miscarried tissue).

43 pregnancies were miscarried <11 gestational weeks (40.2%), 28 pregnancies were lost between 11 and 14 gestational weeks (26.2%), and 36 survived >14 gestational weeks (33.6%). Karyotypes of the tested pregnancies with regard to the gestational age are presented in Table 1. Male karyotype (69,XXY) was present in 62.5% of diandric cases (30/48) and 42.4% of digynic cases (25/59).

**Table 1:** Karyotypes of triploid pregnancies depending on gestational age

Gestational age (gw)	Karyotypes			All *n*
	Female	Male		
	69,XXX *n* (%)	69,XXY *n* (%)	69,XYY *n* (%)	
<11	14 (32.6%)	28 (65.1%)	1 (2.3%)	43
11–14	17 (60.7%)	11 (39.3%)	0	28
>14	21 (58.3%)	15 (41.7%)	0	36

Diandric triploidy was confirmed in 44.9% of all tested samples (48/107; 46.5% (20/43) in pregnancies <11 gestational weeks; 64.3% (18/28) in pregnancies between 11 and 14 gestational weeks; and 27.8% (10/36) in pregnancies which survived >14 gestational weeks).

The distribution of diandric and digynic cases in tested pregnancies with regard to gestational age and tested tissue is presented in Tables 2 and 3, respectively.

**Table 2:** Distribution of diandric and digynic triploid pregnancies depending on gestational age

Gestational age (gw)	Parental origin of triploidy		All *n*
	Diandric *n* (%)	Digynic *n* (%)	
<11	20 (46.5%)	23 (53.5%)	43
11–14	18 (64.3%)	10 (35.7%)	28
>14	10 (27.8%)	26 (72.2%)	36

**Table 3:** Distribution of diandric and digynic triploid pregnancies depending on the method of sample collection

Method of sample collection	Parental origin of triploidy		All *n*
	Diandric *n* (%)	Digynic *n* (%)	
CVS	5 (38.5%)	8 (61.5%)	13
AC	4 (23.5%)	13 (76.5%)	17
D&C	39 (50.6%)	38 (49.4%)	77

*CVS* chorionic villus sampling, *AC* amniocentesis, *D&C* dilation and curettage

## Discussion

Triploidy is a frequent genetic aberration affecting up to 2% of all conceptions [1] and constituting around 10% of chromosomal abnormalities detected in spontaneous miscarriages [17, 18]. Despite the high frequency of this abnormality, the distribution of the diandric and digynic types has not been accurately established to date [1, 4, 7, 9–15].

The identification of the parental origin of triploidy is important as diandric cases are associated with partial hydatidiform mole (PHM) and a slightly increased risk of postmolar gestational trophoblastic neoplasia (GTN) [19, 20]. The risk of GTN after diandric triploid pregnancies with regard to the histopathological features of PHM has not been established to date [21]. Therefore, weekly monitoring of hCG should be implemented after all diandric triploid pregnancies in order to timely diagnose and treat potential neoplasia [22]. Moreover, diandric cases are associated with an increased risk of other maternal complications such as hypertension or hyperthyreosis [23].

There is a broad discrepancy in the literature concerning the prevalence of diandric cases, which according to different authors ranges between 20 and 85%. However, some of the previous studies were affected by selection bias. The high prevalence of diandric triploidy reported by Zaragoza et al. was most probably caused by the preferential inclusion of cases with abnormal placental morphology [12], while the data of Joergensen et al. derive from reference centre for molar pregnancies [15]. In our study, we analyzed prospectively all consecutive triploid samples tested in a single genetic department during a four-year period and report equal proportion of diandric and digynic cases.

Another limitation of previous studies may be an inaccurate determination of the gestational age. As it was reported in earlier studies, diandric triploidy was most frequent in pregnancies miscarried between 10 and 20 gestational weeks [1, 12]. In our opinion, it is more appropriate to divide pregnancies into three groups as it gives the possibility to establish accurately gestational age taking into account the results of routine first-trimester screening. We corrected the gestational age according to the first-trimester ultrasound results and observed a similar frequency of digynic and diandric cases in pregnancy losses before 11 gestational weeks (44.9% of diandric cases), while the highest prevalence of diandric triploidy was observed in the miscarriages between 11 and 14 gestational weeks (64.3%) leading to an evident predominance of digyny in viable second-trimester triploid pregnancies, which is consistent with some earlier studies [7, 10, 11], but in contrary to the others [1, 12–15]. The gestational age of miscarried pregnancies in the study by Jacobs et al. was established only according to the last menstrual period, which could lead to an overestimation of the gestational age and explain the high prevalence of diandric cases in pregnancies lost beyond 14 gestational weeks [1].

Noteworthily, our study is the only one, apart from an old study by Jacobs et al., that includes over 10 triploid cases in all three gestational age groups [1]. This enabled us to establish the distribution of diandric and digynic triploidy with regard to the gestational age.

Similar to our results, a significant decrease in the proportion of diandric cases in pregnancies which survived beyond 14 gestational weeks compared to pregnancies between 11 and 14 weeks (17.9% vs. 47.1%) was observed in a recent study by Lugthart et al., which included mainly triploid pregnancies beyond 11 gestational weeks [7].

A potential limitation of our study is the fact that the samples of miscarried tissue derive from different gynecologic departments and were tested at parental request, while invasive procedures in viable pregnancies were performed in a tertiary referral centre. In our study, triploidy comprised around 10% of all miscarried tissue samples, which is in line with previous studies [17, 18], while in viable first- and second-trimester pregnancies triploidy was detected in 1.4% of cases, which is higher than previously reported [2, 3]. Our department is a tertiary referral centre for high-risk pregnancies, which may explain the higher incidence of triploidy in our population.

In our study, all consecutive triploid cases tested in one genetic department during a four-year period were included. Therefore, we believe that our results most accurately reflect the real distribution of diandric and digynic triploidy both in miscarried samples and samples from invasive prenatal testing.

## Conclusions

Distribution of diandric and digynic triploidy depends on gestational age. The majority of diandric cases are lost in the first trimester of pregnancy. In the second trimester, diandric cases are at least twice less frequent than digynic ones.

## Data Availability

Data available on request from the authors.
